# Optimizing Medical Care during a Nerve Agent Mass Casualty Incident Using Computer Simulation

**DOI:** 10.1007/s10916-024-02094-8

**Published:** 2024-09-05

**Authors:** De Rouck Ruben, Mehdi Benhassine, Debacker Michel, Van Utterbeeck Filip, Dhondt Erwin, Hubloue Ives

**Affiliations:** 1 Research Group on Emergency and Disaster Medicine, Vrije Universiteit Brussel, Laarbeeklaan 103, Jette, 1090 Belgium; 2 Simulation, Modelling, and Analysis of Complex Systems, Department of Mathematics, Royal Military Academy, Renaissancelaan 30, Brussels, 1000 Belgium; 3 Royal Higher Institute for Defence, Renaissancelaan 30, Brussels, 1000 Belgium

**Keywords:** Mass casualty incident, Disaster, Computer simulation, SIMEDIS, Nerve agent, Sarin, GB, Simulation model

## Abstract

**Introduction:**

Chemical mass casualty incidents (MCIs) pose a substantial threat to public health and safety, with the capacity to overwhelm healthcare infrastructure and create societal disorder. Computer simulation systems are becoming an established mechanism to validate these plans due to their versatility, cost-effectiveness and lower susceptibility to ethical problems.

**Methods:**

We created a computer simulation model of an urban subway sarin attack analogous to the 1995 Tokyo sarin incident. We created and combined evacuation, dispersion and victim models with the SIMEDIS computer simulator. We analyzed the effect of several possible approaches such as evacuation policy (‘Scoop and Run’ vs. ‘Stay and Play’), three strategies (on-site decontamination and stabilization, off-site decontamination and stabilization, and on-site stabilization with off-site decontamination), preliminary triage, victim distribution methods, transport supervision skill level, and the effect of search and rescue capacity.

**Results:**

Only evacuation policy, strategy and preliminary triage show significant effects on mortality. The total average mortality ranges from 14.7 deaths in the combination of off-site decontamination and Scoop and Run policy with pretriage, to 24 in the combination of onsite decontamination with the Stay and Play and no pretriage.

**Conclusion:**

Our findings suggest that in a simulated urban chemical MCI, a Stay and Play approach with on-site decontamination will lead to worse outcomes than a Scoop and Run approach with hospital-based decontamination. Quick transport of victims in combination with on-site antidote administration has the potential to save the most lives, due to faster hospital arrival for definitive care.

**Supplementary Information:**

The online version contains supplementary material available at 10.1007/s10916-024-02094-8.

## Introduction

Chemical mass casualty incidents (MCIs) pose a substantial threat to public health and safety, with the capacity to overwhelm healthcare infrastructure and create societal disorder [1]. Nerve agents are particularly alarming in this context due to their high toxicity and potential for fatalities [2]. A comprehensive approach to disaster preparedness and response is paramount; it must integrate elements of early detection, rapid decontamination, and timely medical intervention to mitigate the impact of such incidents [3]. Computer simulation offers a cost-effective and versatile platform for testing the efficacy of different response strategies, thereby complementing traditional contingency planning [4].

The creation of contingency plans often relies heavily on expert opinion, which may not fully account for emergent effects or complex medical-logistical interactions. To address these gaps, we developed a computer simulation whose aim is to provide an evidence-based evaluation of current chemical MCI response strategies and offer recommendations for their optimization.

This model aims to answer the following questions:

1. **Advanced Medical Stabilization Location**: Current contingency plans have a designated mobile medical team (MMT) specifically suited for Chemical, Biological, and Radiological, Nuclear and explosive incidents, the CBRNe MMT. This MMT is given the role of stabilizing victims in the warm zone who are awaiting decontamination, referred to as Advanced Medical Stabilization (AMS). Given the limited treatment capacity of Belgium’s sole CBRNe Mobile Medical Team, would using AMS teams attached to hospitals, paired with hospital-based decontamination, be more effective?
2. **Victim Evacuation Policy**: International MCI plans often employ ‘Scoop and Run’ (ScR) or ‘Stay and Play’ (StP) evacuation policies to transfer victims from the incident site to health care facilities. We aim to assess the suitability of these policies in different settings, particularly focusing on whether ScR is a viable alternative to current StP-oriented plans.
3. **Preliminary Triage Impact**: What is the role of preliminary triage (pretriage) in determining the evacuation order from the incident site to a Casualty Collection Point (CCP), and how does it affect overall MCI outcomes [5]?
4. **Transport Supervision**: In an urban MCI setting, is EMT-only transport sufficient for short distances, especially when compared to MMT-supervised transport [6, 7]?
5. **Hospital Allocation**: Should victims be directed to the closest available hospitals, or should a round-robin distribution be implemented to maximize treatment capacity and minimize transport times [8]?
6. **S&R Capacity**: Is the current Search and Rescue (S&R) capacity adequate, and what are the implications of adding or removing Hazardous Materials (HAZMAT) S&R teams?

## Methods

### Scenario

A scenario was developed in collaboration with subject matter experts from the fire brigade, the Belgian Armed Forces, and the Brussels Intercommunal Transport Company (BITC). In this scenario, a nerve agent is released in a busy subway station beneath a populous winter fairground. This scenario is predicated on the 1995 Tokyo sarin attack, during which the nerve agent sarin was disseminated in multiple subway stations by a terrorist cult [9].

Two aerosol-generating devices are strategically positioned proximate to subway exits. Upon the arrival of the subway train, these devices are activated synchronously to release sarin gas. Concurrently, passengers disembark from the train and traverse through the aerosolized toxic clouds to exit the station, while others embark on the train. The impacted individuals manifest a spectrum of symptoms, from minor ocular and respiratory irritation to acute neurological manifestations such as confusion, agitation and seizures. Incapacitated victims prevent the doors of the train from closing, causing a cessation of train movement on that side of the station.

Immediate communication is established with the national 112 emergency dispatch center (HC112) and the BITC, leading to an immediate cessation of subway line operation and the remote opening of turnstiles for expedited egress. The emergent clinical manifestations among multiple individuals precipitate a state of panic, culminating in a secondary, more chaotic, evacuation phase during which additional traumatic injuries occur.

HC112 identifies the surge in call volume and symptomatology indicative of a chemical incident. In response, multiple deployments of ambulance units and MMTs are initiated, along with the HAZMAT component of the fire brigade. The standard ambulance units are staffed with two basic EMTs, while MMTs consist of an emergency physician and an emergency nurse. Given the suspected chemical mass casualty incident, the specialized CBRNe MMT is dispatched alongside HAZMAT teams.

Upon arrival, the response teams delineate the impacted area into hot, warm, and cold zones, corresponding to varying levels of threat and contamination. HAZMAT teams equipped with Personal Protective Equipment (PPE) commence search and rescue operations within the subway station, simultaneously establishing a medical response chain that includes decontamination procedures. Evacuated individuals are directed to a CCP where triage is initially performed by a physician and a nurse, both equipped with PPE. Triage categories are reassessed after each medical intervention or before each transport.

Post-CCP triage and decontamination, the victims are divided into two cohorts. Ambulatory and mildly affected individuals are conveyed via chartered buses to outpatient medical facilities or even discharged to their residences, their condition allowing. Non-ambulatory individuals are integrated into the medical response chain and subsequently transported to a Forward Medical Post (FMP). At the FMP, victims either undergo stabilizing medical intervention prior to hospital transport or are directly conveyed to designated hospitals with in-transit medical care. Upon hospital arrival, definitive treatment for both chemical and traumatic injuries is administered, marking the ending of the simulated scenario for this victim. The simulation ends when all victims either have received final treatment or are deceased.

### Computer Simulator

The SIMEDIS Simulator constitutes a discrete-event simulation model, utilizing the Julia programming language and predicated on the functionalities offered by the SimJulia package [10, 11]. Central to the simulation environment is the victim model, which records each victim’s spatiotemporal location and evolving health state throughout the entire care continuum. Upon initiation, the simulator loads a pre-defined scenario that encompasses an event timeline, a list of victims, resource inventories, and a set of scenario-specific parameters which are included in appendix 1.

The simulator is designed to implement the Belgian Medical Contingency Plan for disasters, incorporating key elements such as an incident site, a CCP, an optional FMP, and hospitals. Victims are transported between these locations with transport durations being dependent upon the mode of transport and calibrated for each simulated scenario.

Victims flow through the simulation pipeline, starting at the incident site and ending at designated medical facilities. The simulator monitors both the geographical location and the health state of each victim. The latter is governed by a computational victim model elaborated upon in subsequent sections of this publication. Medical interventions can be performed at multiple locations —namely, the incident site, the CCP, the FMP, and within ambulance units—and serve to improve the victims’ health states, consequently extending their potential survival duration, either temporarily or indefinitely.

To account for uncertainty in the timelines, stochastic variability is integrated into S&R, treatment and transport durations. This probabilistic approach serves to better approximate real-world uncertainties and contingencies, thereby augmenting the robustness and generalizability of the simulation results [12].

Sarin dispersion.

Sarin was chosen for its historical precedence in both human and animal studies, as well as its occurrence in accidental and intentional exposures. Its relatively low persistence and ease of synthesis make it a plausible choice for non-state actors [13]. Additionally, sarin can be rapidly identified using field testing kits, although real-world scenarios may experience delays in agent identification, potentially leading to secondary contamination and overestimations of the agent’s physicochemical properties [14, 15]. For the purposes of this simulation, we assumed no secondary contamination among healthcare providers and a sufficiency of resources for FMP treatment and antidote administration.

To rigorously evaluate the disaster response chain, case-mix heterogeneity is required. While scenarios predominated by either unsalvageable victims or those with trivial intoxication levels are plausible in real-world situations, such extremes are not the focus of this research question. The quantity of sarin gas released in the subway station was determined with this in mind.

For the quantification of sarin exposure within the subway environment, an analytical Gaussian Puff dispersion Model (GPM) was constructed to yield a credible yet computationally tractable exposure dose [16]. In parallel, an experimentally validated Computational Fluid Dynamics model of the subway station was developed to explore the airflow patterns, particularly the impact of a moving train on local air circulation. The resulting model shows that the windspeed effects of a stopped train on sarin dispersal at the chosen point are limited. The GPM parameters correspond to an estimated 654 g of Sarin being released using an improvised dispersal device at speeds of 2 g per second. This dispersal rate and quantity are well within limits posited by experts of the US Department of Homeland Security and used in a similar study [17]. A more detailed description of the dispersion model and the concentrations achieved over time can be found in Appendix 2.

### Passenger Flow and Injury Assignment

Victims are instantiated at the incident site and navigate their egress from the subway station. To model the evacuation, we use a social force model as implemented in the Vadere open-source framework for pedestrian dynamics [18]. The actual station architecture is replicated to construct a realistic evacuation model, including the dimensions of obstructions such as the train and staircases.

Two distinct evacuation waves are modeled to simulate varied behavioral responses. The initial wave represents an orderly egress consistent with standard operational procedures, while the second wave consists of a disordered evacuation characterized by panic-induced stampeding and consequent crush injuries and asphyxia. For each individual, a time-concentration integral is computed based on their specific evacuation trajectory and exposed time, as well as the chemical cloud generated by the dispersion model. This allows for the calculation of the individualized dose for each victim. During the second, disordered, evacuation wave, victims are subject to crush-induced traumatic injuries. A compendium of such injuries and their respective frequencies was constructed, drawing on retrospective analyses of stampedes found in the literature. These injuries span a spectrum from superficial lacerations and contusions to severe conditions such as traumatic asphyxia and vertebral fractures [19, 20]. Victims who can self-evacuate, do so on their own accord and will have left the station after 3 min. Victims who cannot self-evacuate due to their health state are evacuated by the HAZMAT (S&R) team. This evacuation process is described in more detail in appendix 2.

The mechanical force exerted on each individual during the evacuation process is derived from the amount of overlap between the circles of influence of the evacuees in the social force model [21]. This total sustained force is used as a proxy to hierarchically assign the corresponding injuries.

### Victim Model

For the purposes of this simulation research, a victim health state model was developed, building upon prior foundational models in the field [22–24]. This newly conceptualized health state, henceforth referred to as the Simedis score (SS), incorporates parameters and categories that are consistent with validated trauma scoring systems [25, 26]. The parameter categories incorporated into the SS are as follows: Glasgow Coma Scale (GCS), oxygen saturation, respiratory frequency, heart rate, and systolic blood pressure. These metrics were selected based on their clinical relevance and utility in trauma care:

- **Glasgow Coma Scale (GCS)**: Provides a comprehensive evaluation of a patient’s level of consciousness.
- **Heart Rate**: Serves as an indicator of both the patient’s level of intoxication and their hemodynamic stability.
- **Systolic Blood Pressure**: Functions as a surrogate measure for organ perfusion and, by extension, organ function.
- **Respiratory Rate**: Acts as a diagnostic marker for both the level of dyspnea and metabolic acidosis.
- **Oxygen Saturation**: Included due to its frequent and straightforward measurement in prehospital trauma care, as well as its utility in differentiating between levels of injury severity. For this parameter, categorization was informed by a comprehensive literature review [27].

Through the incorporation of these clinically significant parameters, the SS aims to provide a nuanced and comprehensive assessment of a victim’s health state and it’s need for urgent life-saving interventions. The components and their scoring values used in the SS are visualized in Table 1.

**Table 1:** Overview of the components used in the Simedis score, their categories and corresponding values

Value	Glasgow Coma Scale	Oxygen Saturation	Heart Rate (bpm)	Systolic Blood Pressure (mmHg)	Respiratory Rate (rpm)
4	13–15	90–100%	61–120	> 89	10–29
3	9–12	85–89%	≥ 121	76–89	> 29
2	6–8	80–84%	41–60	50–75	6–9
1	4–5	< 80%	1–40	1–49	1–5
0	3	0	0	0	0

### Traumatic Injuries

Analysis of previously developed traumatic victim models revealed that clinical progression of categorical victim health scores of untreated victims can be modeled using a sigmoid curve. While others have been proposed, we chose to use a generalized Gompertz curve because of its simplicity and history of applications in modeling growth and mortality [28–31]. Using a set of trauma victims from earlier experiments and data available in the literature, we fitted the parameters to the generalized Gompertz curve based on the victim’s Injury Severity Score (ISS) and age [32]. We refer to appendix 2 for a more detailed explanation describing the assumptions and equations used.

### Chemical Injuries

In parallel to the traumatic evolution, we modeled the effects of chemical injury. For this we assume 7 discrete chemical injury profiles, corresponding to increasing levels of intoxication. These range from very mild (injury profile 1) to lethal (injury profile 6), as well as the absence of intoxication (injury profile 0). These injury profiles are assigned corresponding to a victim’s exposure and are based on military models adapted for civilian purposes [33, 34]. Vital signs and symptoms were added to these victims based on expert opinion in conjunction with existing animal and human experimental data as well as pharmacokinetic/pharmacodynamic models available in the literature [35, 36].

### Triage

As mentioned previously, triage is performed by a triage physician or nurse wearing adequate protection. Triage is based on the NATO triage system for chemical injuries and the SALT triage method for traumatic injuries [37, 38]. The exact implementation is described in appendix 2. For immobile victims, triage is assumed to take 30 s, and 5 s for walking victims. A victim receives both a chemical and a traumatic triage category, and the highest (most serious) triage category of either determines the final triage category. This triage category determines decontamination, treatment and evacuation order. In the case of identical triage categories between victims, the SS is used as a tie breaker.

### Decontamination and AMS

Decontamination is implemented as a time-delay in parallel lanes and includes disrobing, dry padding with absorbent materials and then re-robing. The decontamination and AMS times are based on internal experiments performed at a tertiary hospital in Brussels, which are comparable with times reported in the literature [39, 40]. Mobile victims self-decontaminate, and immobile victims get decontaminated by the firefighters.

An AMS team is present to stabilize severely injured and contaminated victims. Their location depends on the strategy. They identify the victim with the highest triage category, remove it from their waiting queue and administer a limited stabilizing treatment. This treatment consists of antidotes, as well as anticonvulsive and respiratory support if necessary. After their intervention the victim returns to the transport or decontamination queue it was originally in, at the highest priority position.

### Treatment

Treatment effects can be modeled as improvements over time that slow the deterioration or improve the victim’s SS. This improvement can be either temporary or permanent. The act of treatment has a duration based on the triage category and ISS, and the effect of the treatment is based on the provider’s skill level. The duration and effect of the treatment is described in appendix 2. Treatment takes effect instantly, therefore contact of a victim with a treatment provider instantly prolongs the survival time.

### Variable Parameters

#### Pretriage

Preliminary triage (pretriage) is a limited triage that is performed in the subway station to determine the order of Search and Rescue. Victims receive either a yellow (low priority) or a red (high priority) disk from the HAZMAT team members. Pretriage differs from the actual triage which takes place at the CCP, is performed by doctors and nurses, and determines treatment and evacuation order. If pretriage is set to true, the order by which victims arrive at the CCP is determined by the SS at time of contact with the pretriage team. In the case of a tie, a First-In-First-out principle is used. When no pretriage is carried out the victim arrival list is randomized.

#### Strategies

**Fig. 1:**
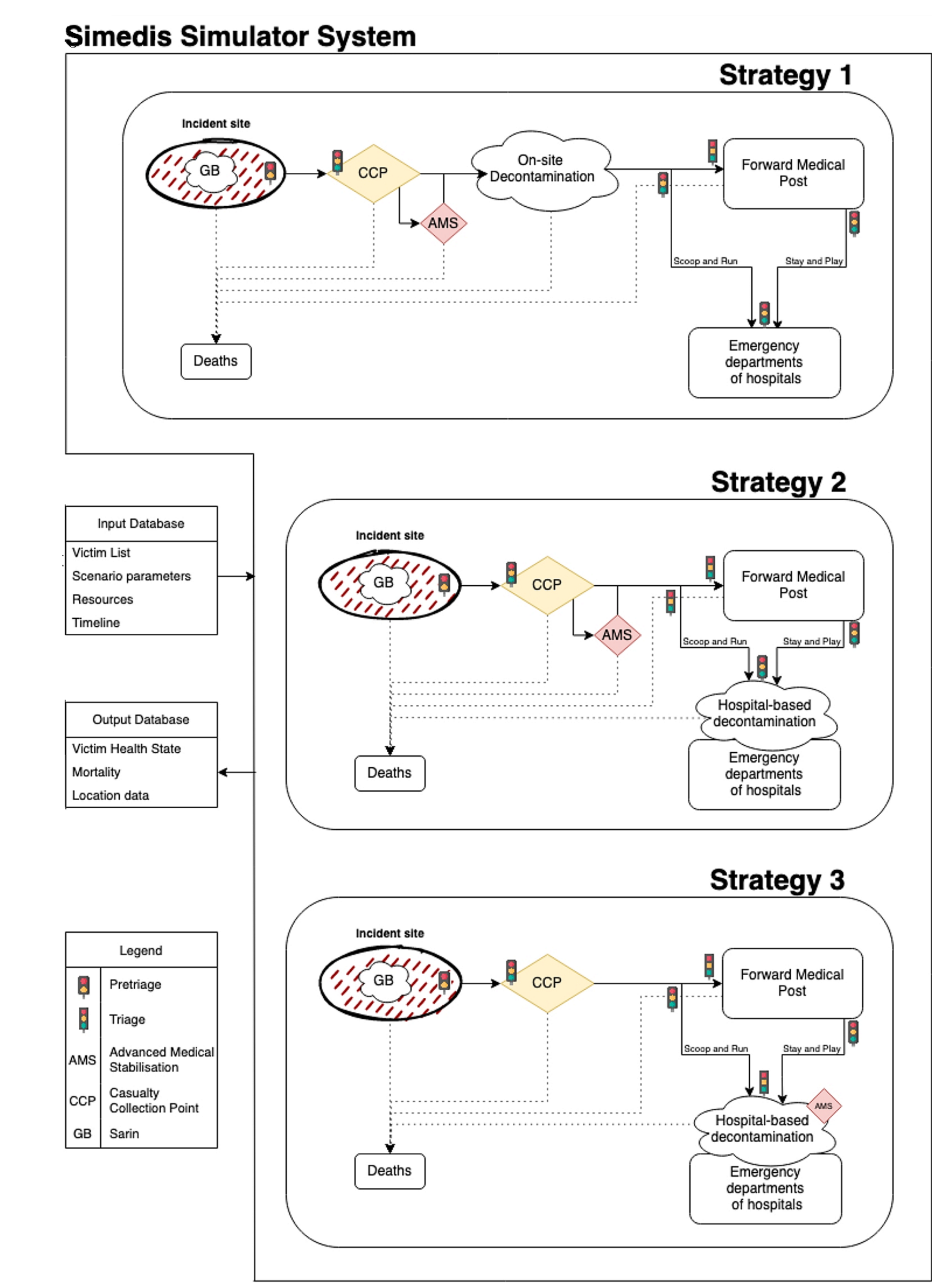
Schematic overview of the victim path throughout simulated 3 strategies. Victims start at the incident site and end up either dead or in the emergency departments of the hospitals. There are three strategies simulated and both include the ‘Scoop and Run’ and ‘Stay and Play’ evacuation policies. The traffic lights represent a point of triage where the triage category of a victim might change. The smaller traffic light with only yellow and red lights represents the pretriage location

Three possible strategies were developed, as depicted in Fig. 1. Strategy 1 is the current strategy with on-site decontamination and AMS in the CCP. Strategy 2 and 3 are novel strategies where contaminated victims receive their treatment and transport by PPE-donned healthcare workers, and decontamination is decentralized and organized by the hospitals themselves. Strategy 2 assumes 1 specific CBRNe MMT who performs on-site warm zone stabilization. Strategy 3 assumes no such MMT but assumes that every hospital has a specific team available at their decontamination unit to stabilize the patients before decontamination commences. In strategy 2 and 3, the treatment time is increased by 50% to account for the difficulties inherent in treating victims while wearing level B PPE [41, 42]. The AMS treatment time is based on real-life measurements from exercises and therefore already adjusted for PPE.

#### Evacuation Policies

There are two main policies implemented in the simulation model as described above: Scoop and Run and Stay and Play. These policies are simulated for every strategy.

#### Search and Rescue Capacity

We assume that all mobile victims left the station and either fled (wild evacuees) or are waiting to be examined at the top of the station at the CCP. Search and rescue at the subway station is performed by the HAZMAT teams of the Fire Service, who are the only personnel permitted to enter the contaminated station to evacuate incapacitated victims. Immobile victims arrive at the CCP based on a fixed inter-arrival rate of 5 min per S&R team consisting of 2 firefighters and a stretcher who perform the search and rescue procedure. There are 3 categories of S&R capacity: low (3 teams), medium (4 teams) and high (6 teams).

#### Patient Supervision during Transport

During the transport phase, patients may be supervised in one of two ways: either by a pair of EMTs or by an MMT.

In the Belgian EMS system EMTs are limited to basic interventions such as oxygen administration, basic splinting and wound care. They are not allowed to administer IV fluids or drugs. Mobile Medical Teams are teams composed of an emergency physician and emergency nurse and can perform almost all advanced life support procedures on the scene. The Belgian EMS is designed to work in a tiered way, according to the needed level of care. A more detailed explanation of the Belgian EMS system can be found in appendix 2.

Two categories of transport supervision for T1/T2 patients have been implemented in the simulation:

- **EMT Supervision**: Characterized by EMT-led supervision, this setting restricts the range of possible medical interventions during transport, thereby attenuating the overall treatment impact, as elaborated upon in the preceding treatment section.
- **MMT Supervision**: Represents the normal HC112 operation standard where an MMT supervises the transport of seriously injured or ill patients.

#### Hospital Allocation

The simulation includes all hospitals located within a one-hour transport time radius. Each hospital’s capacity to receive T1 and T2 victims per hour—known as “surge capacity”—is self-reported and extracted from the hospital contingency plan. Furthermore, the specific medical capabilities of each hospital, such as neurosurgery, cardiothoracic surgery, and Level 1 trauma centers, are integrated into the hospital assignment algorithm. Some hospitals exclusively treat pediatric cases, while others possess distinct pediatric treatment capacities.

Prior to hospital transport, the Simedis score (SS) is evaluated to estimate each victim’s probability of surviving the transport. Victims with an SS less than 5 are presumed unlikely to survive transport. Two distinct hospital distribution policies have been implemented:

- **Closest First Policy**: Prioritizes filling available hospital capacity based on their proximity to the incident site.
- **Spread Out Policy**: Aims to distribute victims evenly across multiple hospitals in a round robin manner, thereby minimizing resource downtime at individual healthcare facilities.

### Statistical Analysis

A sensitivity analysis determined that 10 simulator replications are adequate to get significant results. We created an ordinary least squares linear regression model to analyze the effect of every separate parameter on the prehospital mortality which is our primary outcome variable. The analysis was performed with the python package statsmodels [43].

## Results

**Fig. 2:**
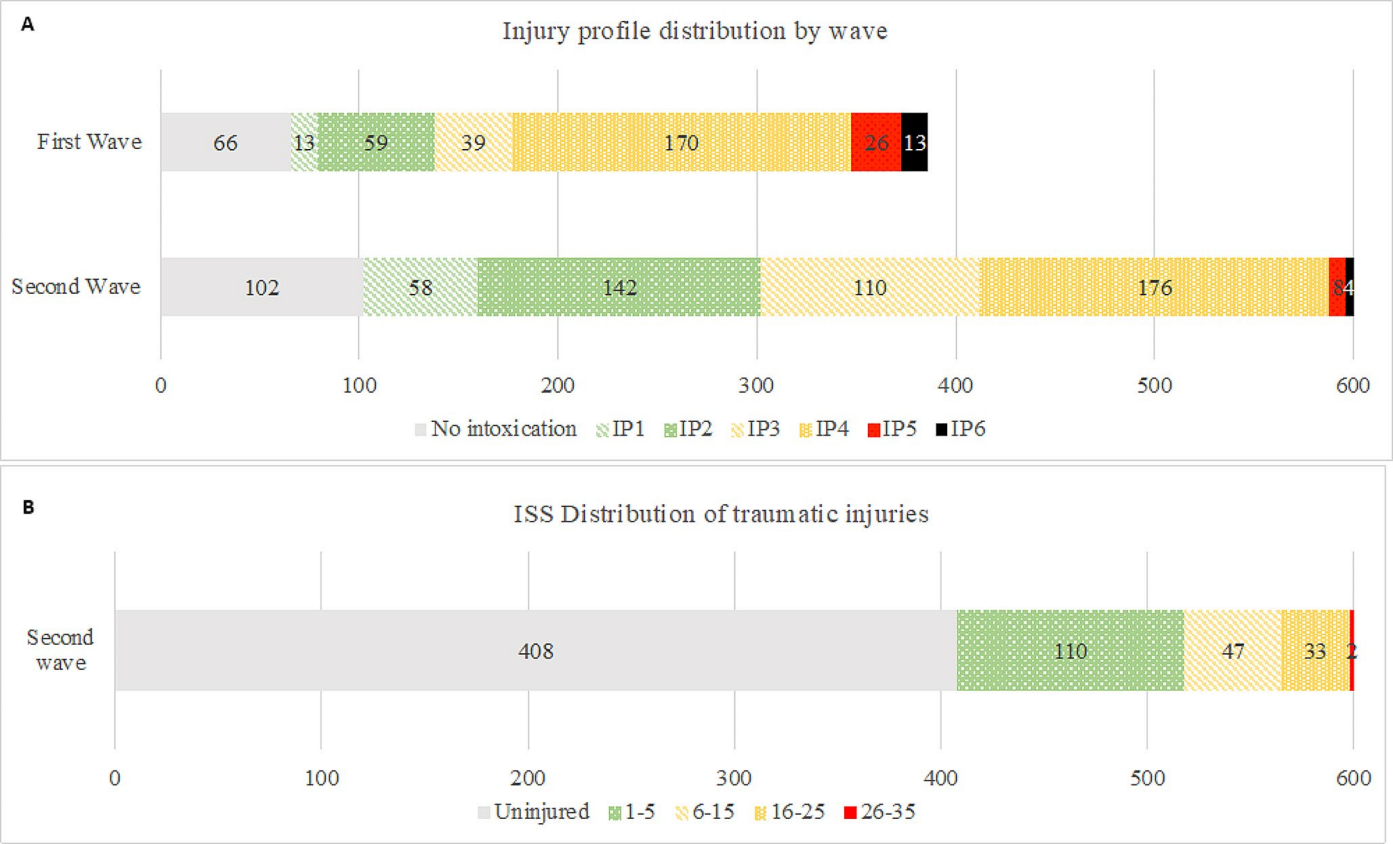
(**A**) Victim’s chemical injury profile distribution by subway evacuation wave. (**B**) Victim’s injury severity distribution by ISS

Following consultations with experts from the BITC and leveraging data on the mean subway train occupancy during evening peak hours, the simulation commenced with an initial cohort of 986 victims. Of these, 386 individuals evacuate in the first wave, leaving 600 for the second wave. Across both evacuation phases, only 168 individuals exhibited no detectable levels of intoxication. Within the tumultuous environment of the second evacuation wave, 192 victims incurred injuries, among which 110 were classified as minor (ISS < 6). Tables 2 and 3, along with their complementary Fig. 2A and B show the distribution of both chemical and traumatic injuries over both waves.

**Table 2:** Resultant chemical injury profile count per wave

	None	IP1	IP2	IP3	IP4	IP5	IP6	Total
First Wave	66	13	59	39	170	26	13	386
Second Wave	102	58	142	110	176	8	4	600
Totals	168	71	201	149	346	34	17	986

**Table 3:** Resultant injury severity score count per of the second evacuation wave

	Uninjured	1–5	6–15	16–25	26–35	Total
Second Wave	408	110	47	33	2	600

The results of the linear regression analysis are displayed in Table 4. This analysis has an R-squared value of 0.836 and an adjusted R-squared value of 0.835. The F-statistic of the regression analysis is 729.5 and is significant (*p* < 0.001). Statistically significant predictors of mortality are the strategy and policy used (*p* < 0.001), as well as pretriage (*p* = 0.046). The other variables failed to overcome the significance threshold. The Omnibus probability value of 0.008 and Jarque-Bera probability value of 0.005 indicate non-normality in the residuals. A robust linear model was constructed with similar coefficients and *p*-values, indicating that these non-normal residuals have little effect on the validity of the analysis.

**Fig. 3:**
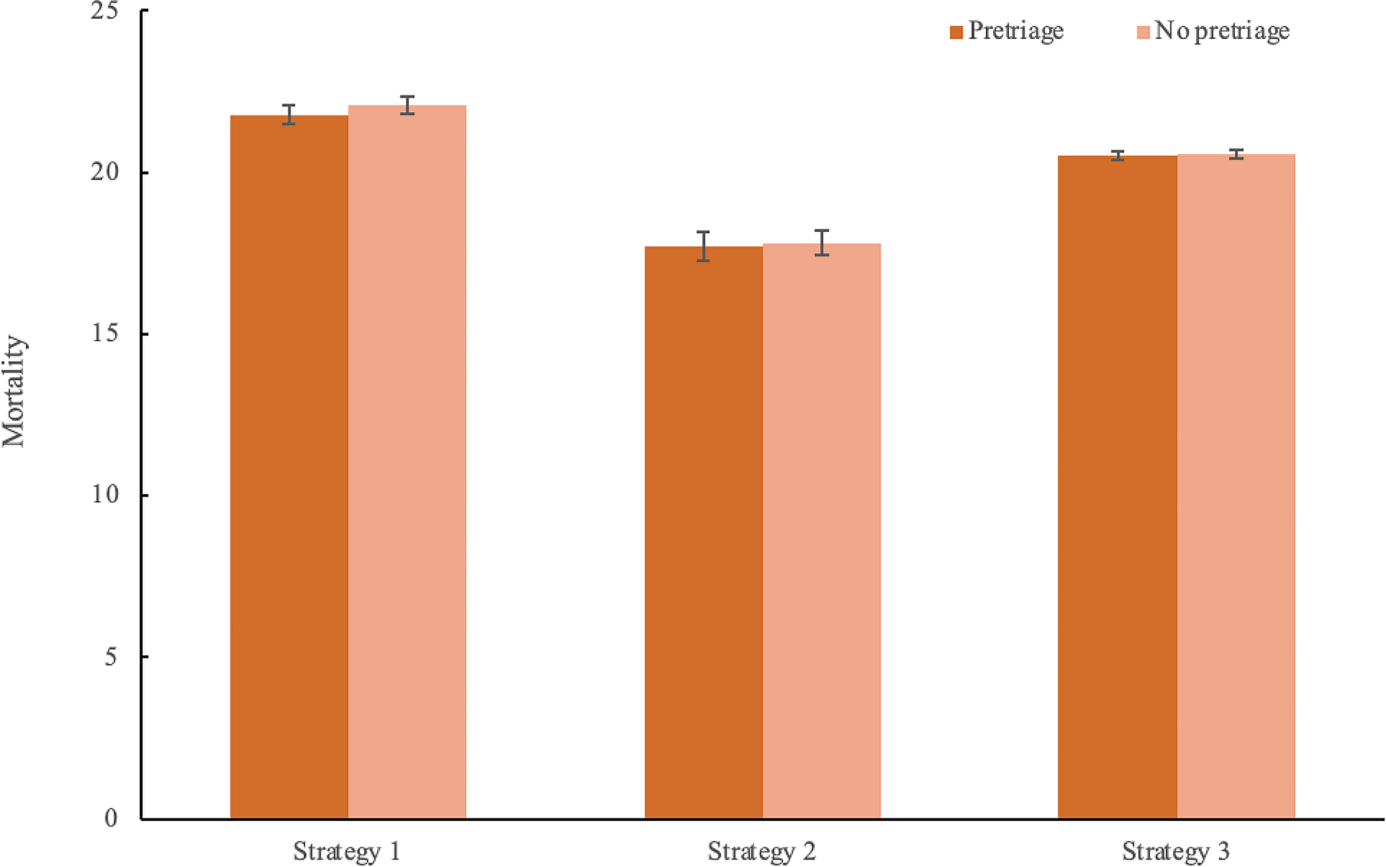
Prehospital mortality for the different strategies by pretriage. The error bars indicate 95% confidence intervals

**Fig. 4:**
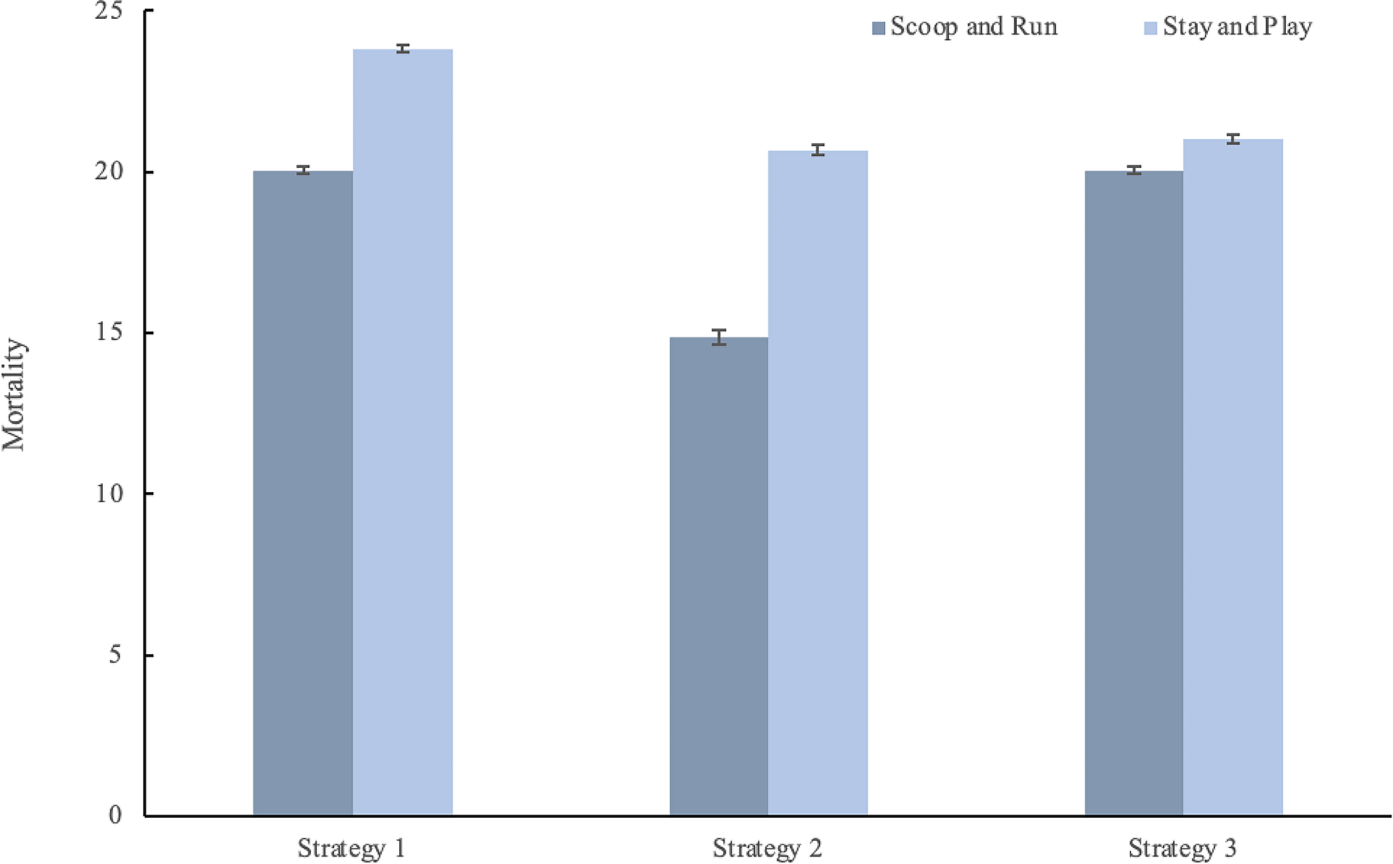
Prehospital mortality for the different strategies by evacuation policy. The error bars indicate 95% confidence intervals

In this simulation exercise, both supervision level and hospital distribution failed to show a significant difference between the categories simulated. We observed no difference between the simulated search and rescue rates. Figures 3 and 4 give a graphic representation of the results in Table 5.

**Table 4:** Results of the linear model analysis. Statistically significant predictors are marked in bold

	coef	std err	*p*-value	confidence interval	
**Intercept**	**15.97**	**0.122**	**< 0.001**	**15.727**	**16.207**
Search and Rescue rate (Low)	1.1e-14	0.100	1.000	-0.196	0.196
Search and Rescue rate (Medium)	8.9e-15	0.100	1.000	-0.196	0.196
Transport supervision (MMT)	0.121	0.082	0.139	-0.039	0.281
Hospital distribution (Spread Out)	0.104	0.082	0.202	-0.056	0.264
**Strategy 1**	**4.175**	**0.100**	**< 0.001**	**3.979**	**4.371**
**Strategy 3**	**2.781**	**0.100**	**< 0.001**	**2.585**	**2.977**
**PreTriage (Yes)**	**-0.163**	**0.082**	**0.046**	**-0.322**	**-0.003**
**Policy (Stay and Play)**	**3.529**	**0.082**	**< 0.001**	**3.369**	**3**

The total average mortality ranges from 14.7 in the combination of strategy 2 Scoop and Run policy with pretriage, to 24 in the combination of Strategy 1 with Stay and play and no pretriage. The average mortality per strategy, policy and pretriage configuration is displayed in Table 4.

**Table 5:** Descriptive statistics of the (significant) results

Strategy	Policy	Pretriage	Mean	Std dev	Min	Max
Strategy 1	ScoopRun	No	20.2	0.88	19	21
		Yes	19.9	0.70	19	21
	StayPlay	No	24.0	0.69	23	25
		Yes	23.7	1.22	22	25
Strategy 2	ScoopRun	No	15.0	1.56	12	17
		Yes	14.7	2.01	11	18
	StayPlay	No	20.7	1.18	19	23
		Yes	20.7	1.44	19	23
Strategy 3	ScoopRun	No	20.2	0.88	19	21
		Yes	19.9	0.70	19	21
	StayPlay	No	21.0	1.08	19	23
		Yes	21.1	1.13	20	23

## Discussion

### Should Decontamination and/or AMS Teams be Located at the Scene or at Hospitals?

The results suggests that Strategies 2 and 3, which involve either on-site AMS and hospital-based decontamination or both AMS and decontamination at the hospital, respectively, yield lower mortality rates compared to the current Belgian standard of on-site AMS and decontamination (Strategy 1). This indicates that moving decontamination activities near to or into the hospital setting could improve survival rates. The decision to move AMS to the hospital setting depends on the chosen evacuation policy but can have a major impact.

Strategies 2 and 3 were introduced as alternatives to mitigate the logistical challenges associated with antidotes, PPE and decontamination equipment. These strategies posit decentralized repositories situated at secure locations close to the point of need but require levels of preparedness not yet achieved [44]. While these approaches may necessitate additional training and materials expenditures, they would effectively ameliorate on-site bottlenecks and improve patient outcomes. They also afford greater operational flexibility, including the ability to dynamically respond to evolving threats or unsafe operational environments. Noteworthy limitations include the potential for ambulance contamination, the implications of which are assumed to be negligible in this study, and uncertainties regarding the ability to decontaminate exposed materials [45].

### Scoop and Run versus Stay and Play

The “Scoop and Run” approach, which is more conducive to Strategies 2 and 3, shows a marked benefit in reducing prehospital mortality. We explain this by the fact that the FMP takes a long time to set up, and during this time several victims die before they can even start to receive treatment. The proximity of a high number of healthcare facilities to the incident site, in combination with the limited EMT treatment which marginally increases survival time makes ScR the more successful strategy in this scenario. This finding is consistent with previous simulation experiments [23, 46].

### What is the Effect of Preliminary Triage?

Unlike prior simulations which focused on scenarios with only traumatic injuries, the pretriage parameter in this study exerts negligible influence. The specific victim demographics and the implementation of pretriage result in only minor variations in victim health states at the time of pretriage assessment, thereby minimally affecting the evacuation list sequencing. Within the simulator, victims undergo multiple triage assessments, correcting for possible erroneous evacuation ordering. Victims triaged as minor (T3, also known as “green”) are redirected to outpatient treatment centers and exit the simulation, which is also a form of (pre)triage. These multiple layers of triage in combination with the treatment capacity freed seems to compensate for the minor impact of a pretriage mechanism in this scenario.

### What is the Effect of an MMT to Supervise Transport Compared to EMT-only Transport?

Our data did not show a significant impact of MMT transport supervision when compared to EMT-only transport. This finding is in line with the other findings, namely that the proximity of a high number of hospitals with high-level treatment capacities mitigates the decline in the patients’ health state due to inferior treatment during EMT transport, and additional waiting time in the case of MMT transport.

### Should we Fill the Closest Hospitals First or Use a Round-Robin Fashion?

Our data did not show a significant impact of hospital distribution strategy on mortality. We explain this again by the urban environment as previously mentioned.

### Is the S&R Capacity Adequate for this Scenario?

The simulation does not show any significant effect of increasing the number of HAZMAT teams. We assume this is due to the limited number of immobile victims and the timing of the delay of antidote administration until contact with the AMS team.

### General Considerations and Limitations

Analysis of simulation results indicates that a substantial proportion of mortality occurs prior to the intervention of the AMS team. These mortalities are predominantly associated with the combination of high-level chemical injuries and moderate-to-severe traumatic injuries. This leads to a baseline of 17 victims considered non-salvageable under current conditions. However, the timely administration of antidotes, potentially during the S&R phase, could convert these otherwise non-salvageable cases into treatable ones, and have a significant impact on the mortality.

In our simulation, prehospital mortality serves as the sole outcome metric and is a binary metric. The simulator’s current configuration lacks the power to allow estimation of medium- and long-term outcomes. This limitation stems from inherent uncertainties within the victim model. Continued research is warranted to examine both short-term and long-term outcomes, particularly in the context of concomitant chemical and traumatic injuries.

We have purposefully opted to exclude mass psychogenic illness (MPI) from our simulation model. Mass Psychogenic illness is a culture- and zeitgeist-dependent phenomenon with a unclear definition and uncertain cause, not necessarily requiring proximity to an MCI [47]. Despite this, MPI is reportedly present in a significant minority of chemical incidents [48, 49]. Our specific aim of the simulation model is to enhance response effectiveness to verifiable chemical casualties. The exclusion of MPI is based on precedent methodologies such as those used in the Tokyo sarin attack, where ocular symptoms were a primary diagnostic tool to differentiate affected individuals from the worried well [50, 51]. Our assumption is that these individuals are referred to alternative healthcare facilities or specifically designated areas of healthcare facilities [52]. However, this assumption could oversimplify the real-world dynamics and impact on emergency services. Our study also does not consider the resource allocation for MPI, nor does it propose strategies to manage or mitigate its effects post-incident. This aspect could be explored in future research to fully understand the scope and impact of MPI in chemical terrorist attacks. Potential adjustments to model include non-ambulance transport, self-referral processes and worried well taking up space in the hospital response chain.

The simulation model presupposes a degree of timeliness and organization that is often unattainable in real-world MCIs, which are frequently characterized by disorder and operational challenges [53]. We also assumed no secondary contamination among healthcare providers and a sufficiency of resources for FMP treatment and antidote administration, assumptions that may not hold true in real-world situations. Future research could explore the incorporation of communication dynamics and individual interactions among healthcare workers, patients, and medical directors. Such enhancements would aim to simulate the chaotic nature of incident sites more authentically.

Finally, we would like to mention that the computational intensity of the fluid dynamics model for the subway station necessitated the assumption of halted subway line circulation within the scenario. Incorporating the piston effect of multiple train arrivals would likely increase in the spread of Sarin, increasing the number of victims [54].

## Conclusion

In this paper, we presented the results of a computer simulation model designed to test local contingency plans for a chemical mass casualty incident. We formulated a simulation model to address a series of questions pertinent to chemical mass casualty care, framed within a plausible scenario that is relevant to the organization of both prehospital and hospital-based care. The model integrates existing physicochemical models, crowd evacuation models, and medical victim models within a discrete event simulation framework, thereby providing a comprehensive and realistic computer simulation model.

Our findings suggest that in this specific simulated urban chemical mass casualty incident, the conventional strategy involving an FMP and prehospital decontamination leads to suboptimal outcomes compared to a “Scoop and Run” approach with hospital-based decontamination. The data indicate that expedited transport of victims, accompanied by prehospital administration of antidotes, holds the potential for maximal life preservation. This can be attributed to reduced bottlenecks at the incident site, resulting in enhanced transport efficiency and accelerated arrival at hospitals for definitive care. Furthermore, this strategy manifests significant resilience in the context of a terrorist attack, permitting more rapid scene clearance and dynamic rerouting of resources.

While useful, this model lacks granularity and may not fully capture some of the nuanced interactions between different types of injuries and treatment approaches. Moreover, the operational realism of the simulation is limited by the discrepancy between the organized nature of simulation environments and the often chaotic circumstances of real-world mass casualty incidents. These limitations suggest that future simulation models should incorporate the dynamics of inter-agency communication and decision-making processes.

In summary, our study not only affirms the role of computer simulation as a potent instrument in the disaster preparedness arsenal but also highlights its limitations and avenues for future research. While each decision simulated has its inherent drawbacks, advantages, and limitations, we believe that our computer simulation model serves as a valuable tool for estimating their probable impact on patient outcomes. Advancements in simulation techniques and subsequent research could contribute substantively to the optimization of strategies and policies for managing complex mass casualty incidents involving both chemical and traumatic injuries.

## Electronic Supplementary Material

Below is the link to the electronic supplementary material.

Supplementary Material 1

Supplementary Material 2

Supplementary Material 3

## Data Availability

No datasets were generated or analysed during the current study.
