# Supplementary material for: Optimizing Medical Care during a Nerve Agent Mass Casualty Incident Using Computer Simulation
Source: J Med Syst. 2024 Sep 5;48(1):82. doi: 10.1007/s10916-024-02094-8 (PMC11377464; doi:10.1007/s10916-024-02094-8)
Supplement: Supplementary file 1 — Supplementary Material 1 [file 10916_2024_2094_MOESM1_ESM.docx]

Appendix 1 Simulator Settings

Supplementary information to the article ‘Optimizing Medical Care during a Nerve Agent Mass Casualty Incident using Computer Simulation’ in Journal of Medical Systems

**Authors:**

De Rouck Ruben, MD (1)

Mehdi Benhassine, PhD (2)

Debacker Michel, MD (1)

Van Utterbeeck Filip, PhD (2)

Dhondt Erwin, MD (3)

Hubloue Ives, MD, PhD (1)

Corresponding Author: Ruben De Rouck – [ruben.de.rouck@vub.be](mailto:ruben.de.rouck@vub.be)

**Author Affiliations**

1. Research Group on Emergency and Disaster Medicine, Vrije Universiteit Brussel, Laarbeeklaan 103, 1090 Jette, Belgium
2. Department of Mathematics, Royal Military Academy, Renaissancelaan 30, 1000 Brussels, Belgium
3. DO Consultancy, Brussels, Belgium

Speed Variables: transport time is distance divided by speed.

1. cityspeedAMB = 20: Average ambulance speed in the city, measured in km/h.
2. roadspeedAMB = 45: Average ambulance speed on roads, measured in km/h.
3. highwayspeedAMB = 90: Average ambulance speed on highways, measured in km/h.
4. cityspeedMMT = 35: Average MMT speed in the city, measured in km/h.
5. roadspeedMMT = 70: Average MMT speed on roads, measured in km/h.
6. highwayspeedMMT = 120: Average MMT speed on highways, measured in km/h.

Personnel and Resource Variables

1. minNurseFMP = 3: Minimum number of nurses required at the FMP.
2. minDoctorFMP = 2: Minimum number of doctors required at the FMP.
3. nrTriageMMT = 2: Number of MMTs reserved for triage operations.

Time Variables

1. evacTimeT3 = 5.0: Time in minutes after sarin release for evacuating T3 victims and uninjured individuals.
2. SRArrivalLowerBound = 15.0: Lower bound for firefighter arrival time, in minutes.
3. SRArrivalUpperBound = 20.0: Upper bound for firefighter arrival time, in minutes.
4. setupFMPtime = 35: Time required to set up the FMP, in minutes.
5. TotalSmallNoriaTransportTime = 4.6: Time for transporting victims from CCP to FMP, in minutes.
6. UnloadHosp = 2: Time to unload victims from the ambulance at the hospital, in minutes.
7. HospDropOff = 14: Time for ambulance restocking and briefing at the hospital, in minutes.
8. TriageTimeT3 = 0.08: Time for T3 triage, in minutes.
9. PreTriageTime = 0.5: Time for pretriage per victim, in minutes.
10. TriageTimeUrgentDefault = 0.5: Default time for urgent triage per victim, in minutes.

Search and Rescue (S&R) Variables

1. srInterval = 5.0: Inter-arrival rate for evacuation of victims, in minutes.
2. srCapacityLowStart = 2: Initial SR team capacity for "Low" rate.
3. srCapacityMediumStart = 3: Initial SR team capacity for "Medium" rate.
4. srCapacityHighStart = 4: Initial SR team capacity for "High" rate.
5. srCapacityLowExtra = 1: Additional SR team capacity for "Low" rate.
6. srCapacityMediumExtra = 1: Additional SR team capacity for "Medium" rate.
7. srCapacityHighExtra = 2: Additional SR team capacity for "High" rate.

Decontamination and AMS Time Variables

1. disrobetimes = (2.0,1.0): Time for disrobing, in minutes.
2. passtimes = (1.0,0.33): Time for passing, in minutes.
3. decontimes = (2.33,0.75): Time for decontamination, in minutes.
4. rerobetimes = (2.4,1.2): Time for rerobing, in minutes.
5. amst_arrivaltime = 20.0: Time for AMS team arrival, in minutes.

Stochastic Variability Variables

1. sigmaLN = 0.5: Shape parameter for LogNormal distribution.
2. sigmaN = 3: Number of sigma equal to boundary for Normal distribution.
3. TreatTimeDist = "Triangular": Distribution type for treatment time.
4. TreatTimeBound = 0.2: Allowed deviation for treatment time.
5. TrigTimeDist = "Triangular": Distribution type for trigger time.
6. TrigTimeBound = 0.2: Allowed deviation for trigger time.
7. TravTimeDist = "LogNormal": Distribution type for travel time.
8. TravTimeBound = 0.25: Allowed deviation for travel time.
9. RescueTimeDist = "Normal": Distribution type for rescue time.
10. RescueTimeBound = 0.25: Allowed deviation for rescue time.
11. OtherTimeDist = "Normal": Distribution type for other times.
12. OtherTimeBound = 0.2: Allowed deviation for other times.
