# Supplementary material for: Optimizing Medical Care during a Nerve Agent Mass Casualty Incident Using Computer Simulation
Source: J Med Syst. 2024 Sep 5;48(1):82. doi: 10.1007/s10916-024-02094-8 (PMC11377464; doi:10.1007/s10916-024-02094-8)
Supplement: Supplementary file 2 — Supplementary Material 2 [file 10916_2024_2094_MOESM2_ESM.docx]

Appendix 2: Detailed description of the SIMEDIS Simulator

Supplementary information to the article ‘Optimizing Medical Care during a Nerve Agent Mass Casualty Incident using Computer Simulation’ in Journal of Medical Systems

**Authors:**

De Rouck Ruben, MD (1)

Mehdi Benhassine, PhD (2)

Debacker Michel, MD (1)

Van Utterbeeck Filip, PhD (2)

Dhondt Erwin, MD (3)

Hubloue Ives, MD, PhD (1)

Corresponding Author: Ruben De Rouck – [ruben.de.rouck@vub.be](mailto:ruben.de.rouck@vub.be)

**Author Affiliations**

1. Research Group on Emergency and Disaster Medicine, Vrije Universiteit Brussel, Laarbeeklaan 103, 1090 Jette, Belgium
2. Department of Mathematics, Royal Military Academy, Renaissancelaan 30, 1000 Brussels, Belgium
3. DO Consultancy, Brussels, Belgium

This appendix contains a more detailed description of the SIMEDIS simulator including information on the Belgian EMS system, the sarin dispersion in the subway station, the injury assignment of the victims, the clinical evolution of their health state, the S&R modelling, the triage algorithm, and the impact of treatment on the health state of the victims.

1. Belgian Emergency Medical Services system

The Belgian Emergency Medical Services (EMS) system operates on a comprehensive structure designed to provide varying levels of medical care to patients in emergency situations. The system is primarily activated through the emergency number 112, which serves as the centralized contact point for medical, fire, and police services. Upon receiving a call, the dispatch center assesses the nature and severity of the emergency to allocate appropriate resources. The Belgian EMS system offers a tiered approach to emergency medical care, ranging from basic interventions by regular ambulances, via more advanced interventions by paramedic intervention teams (PIT) to advanced lifesaving interventions by mobile medical teams (MMT). This structure ensures that patients receive the most appropriate level of medical attention, tailored to the severity of their condition.

Regular Ambulances

Regular ambulances form the backbone of the EMS system and are generally the first to be dispatched to the scene of the emergency and can solve most minor emergencies. These ambulances are staffed with two emergency medical technicians (EMTs) trained in basic life support (BLS) procedures. They are equipped to manage minor injuries and medical conditions, including blood glucose testing, wound dressing, splinting and administration of oxygen However, EMTs are not authorized to perform advanced medical procedures or administer drugs or IV fluids.

Paramedic Intervention Teams (PIT)

PIT units are an intermediary level of care between regular ambulances and MMTs. Staffed with one or more EMTs and an emergency nurse, PIT units can handle more severe cases that require interventions beyond the scope of BLS. This includes intravenous fluid administration, cardiac monitoring, and the use of certain medications under established protocols. PIT units often act as a bridge when MMTs are unavailable or en route, providing necessary stabilization before higher-level medical care is available.

Mobile Medical Teams (MMT)

MMTs represent the highest level of pre-hospital care in the Belgian EMS system. MMTs are staffed with an emergency physician and emergency nurse, capable of delivering hospital-level care at the scene. They are equipped with advanced medical equipment and a broad range of medications, allowing them to perform complex interventions such as endotracheal intubation, advanced cardiac life support, and even surgical procedures in extreme cases. MMTs are dispatched for the most severe medical emergencies, often involving life-threatening conditions or complex clinical scenarios that necessitate immediate advanced medical interventions.

Each tier is designed to work in synergy, ensuring a seamless transition of care as medical needs escalate. For instance, a regular ambulance may initially respond to a call but can later be supplemented or replaced by PIT or MMT resources as the situation evolves.

In the SIMEDIS simulator most of local and regional MMTs are dispatched to the disaster site to deal with the MCI. In the contingency plan, PITs and a certain number of MMTs are reserved for routine emergency operations. Therefore, no PITs are included in the simulator.

2. Sarin Dispersion

The Computational Fluid Dynamics model by Faugier et al. indicates that while the "piston effect" of a train arriving at a station initially induces high wind speeds, these speeds diminish to only 0.5 m/s once the train becomes stationary [1]. This observation aligns with the dynamics of train-induced airflow; the wind field is significant during the train's movement, peaking as the train arrives, but vanishes when the train halts. This understanding refines the interpretation of Hosseini et al.’s findings, which suggest that the piston effect from train movement significantly spreads sarin to surrounding areas when released into a ventilation system with active train traffic [2]. In our scenario, however, the release of sarin occurs after the train has already stopped, during the period when the wind speeds are decreasing. Therefore, while Hosseini et al.’s insights are valuable, they are less directly applicable to our specific case where train movement has ceased, and we assume all circulation of trains in the subway station is stopped.

The Gaussian puff model predicts that peak sarin concentration in the subway station is reached within 1 minute and quickly decreases as the wave fans out. We assume that the concentration is homogenous over the 1 square meter subsection of the station and calculate the absorption by measuring the time spent inside this segment by the concentration of the segment. Figures 1 A to D shows the evolution of this plume over time after 1, 5, 10 and 20 minutes.

**A) t = 1 min B) t = 5 min**


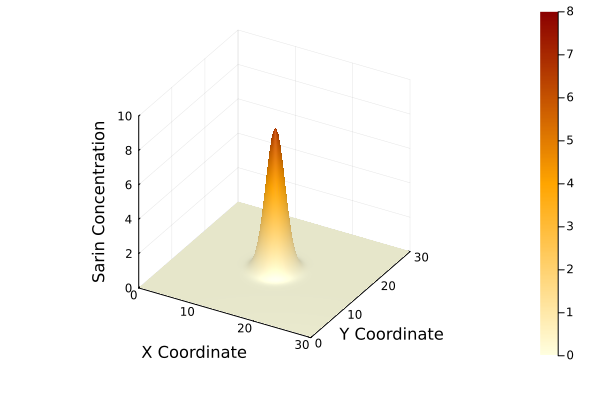

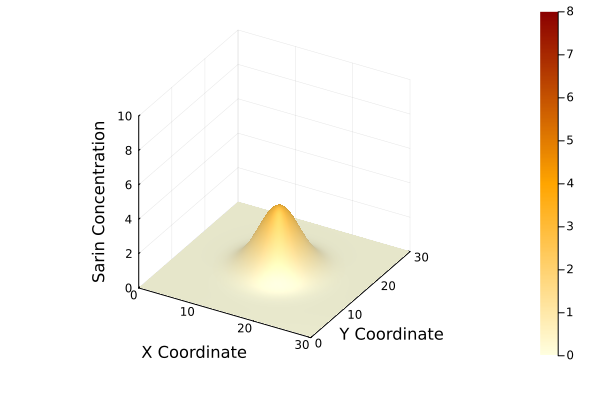


**C) t = 10 min D) t = 20 min**

**
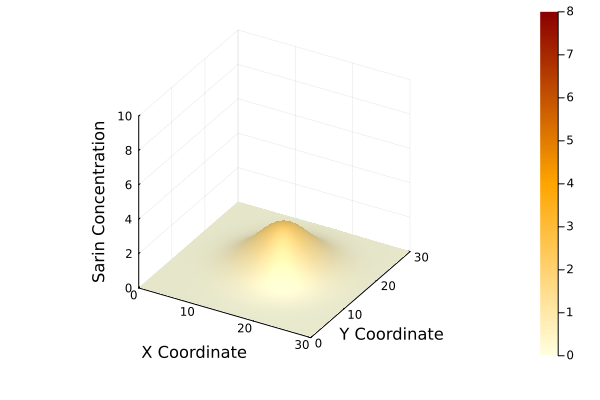

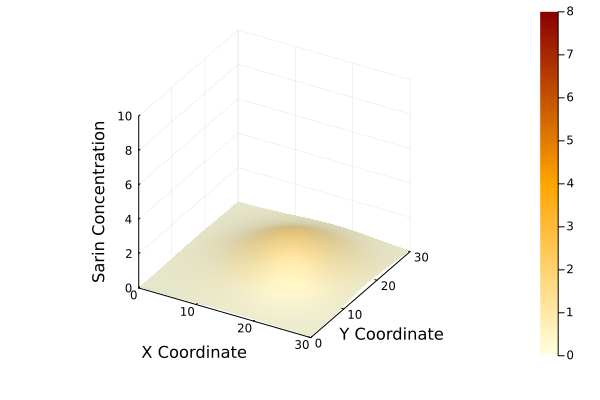
**

**Figure 1: Sarin concentration over time. Sarin concentration is expressed in mg.m^-3^. The center of the map (15,15) corresponds to the point where the sarin is released. Figures A to D represent the cloud concentration at time equals 1 minute after release to time equals 20 minutes after release.**

****3. Clinical evolution of health state****

In the realm of mass-casualty incident simulations, understanding the health state evolution of victims, especially in the context of trauma and chemical injuries, is paramount. This study leverages the Generalized Gompertz function, which is a modification of the function originally proposed by Benjamin Gompertz in 1825, to model the clinical evolution of a victim's health state due to trauma.

The resulting equation describing the evolution of the Simedis score of the traumatic injuries is:

$\mathrm{SS}_{\mathrm{trauma}}\left( t \right)=a-\left( a-ae^{-e^{\left( b-ct \right)}} \right)^{\gamma}$ (1)

Where:

- t is the time elapsed (starting from the time of injury) expressed in minutes.
- a is an asymptotic value, representing the eventual outcome or limit. For this study, a is a constant set to 20 as the sum of the 5 scores corresponding to a healthy individual.
- e is Euler’s number
- b denotes the shift in time, determining when the function starts its decline.
- c is the rate of decrease.
- γ is a positively defined shape parameter, and has the property of not letting the result tend to 0.

This function is particularly adept at capturing the nuances of trauma progression. The relationship between a victim's age and the severity of their injuries is intricately tied to the parameters b, c, and γ

****4. Linking Injury Severity Score to Time of Death****

The Injury Severity Score (ISS) serves as a quantitative measure of the severity of traumatic injuries, derived from the anatomical location and intensity of injuries. Specifically, it is computed from the sum of the squares of the Abbreviated Injury Score (AIS) for the three most severely injured body regions.

Historically, the correlation between ISS and mortality has been a focal point in trauma research. However, a significant portion of this research is grounded in trauma registries, where the observed outcomes are invariably influenced by therapeutic interventions. To gain a clearer understanding of the natural progression without treatment, our approach centered on post-mortem studies of untreated victims. Such studies, which offer insights into the estimated time of death, are notably limited in the literature.

After collating data points from these select studies, we employed an exponential function for fitting. Potential limitations of this approach are AIS inter-observer variability, the fact that it only considers 1 injury per anatomical region and the uncertainty on the margin of error of the inferred time of death from the studies. The complete methodology and references used is described in Benhassine et al [3].

The relationship between the ISS and the time of death, t_death_​, is given by:

t_death_ = 43500 ISS^(-1.95)^ (2)

****5. Determination of Gompertz Curve Parameters****

The γ parameter plays a significant role in determining the outcome of the victim. For instance, the γ parameter is set to 1 only if at least one lethal injury is present. This ensures that the function approaches zero at a time t_death_ which is approximately (b−e)/c, where e stands for Euler’s number.

The parameters b, c, and γ are intricately tied to the victim's age and the severity of their injuries. The function, when γ=1, will approach zero at a time t_death_​ of approximately (b−e). If the ISS is smaller or equal to 25, the value of b is determined by the patient's age, with different equations applied for different age ranges.

The b parameter is calculated as follows:

1. **For Young Patients (Age 12 or Below)**: b = 0.0055*patient.age - 0.09
2. **For Elderly Patients (Age Above 70)**: b = -0.0067*patient.age +0.4433
3. **For Middle-Aged Patients (Age Between 12 and 70)**: b = -0.03.

If the ISS is 26 or greater, then the equations for b are adjusted by subtracting 0.04 from each equation.

6. Chemical injuries

Chemical injuries introduce another layer of complexity. While the ISS provides a robust framework for physical injuries, chemical injuries require an adjustment to equation one. To create a mixed chemical and traumatic model, we used formula 3 where the Delta represents the difference from the maximum SS of 20.

SS(t) = 20 – ΔSS_trauma_(t) – ΔSS_chemical_(t) (3)

Here, ΔSS _chemical_(t) is a function that encapsulates the health state deterioration after the inhalation of a toxic chemical agent, with sarin (GB) being the primary agent of interest in this study. A continuous function was fitted to convert the discrete profiles to a continuous evolution. We found the Chi Square function to be the best fitting function for our dataset.

We want to stress that this is a very simplified combination model, based on the assumption that the injuries don’t interact and additively increase the deterioration speed. This assumption was deemed necessary due to the limited data available at this moment to quantify the interaction and might need to be updated when more data becomes available. Further investigation is imperative to improve modeling of potential interactions of chemical and/or multiple traumatic injuries and the impact these interactions have on their respective treatments.

7. Search and Rescue modeling

A patient’s mobility is defined by either a total Simedis score less than 17, or linked to specific injuries that impact mobility such as ankle fractures or severe hemorrhage. Victims who are classified as immobile and are unable to self-evacuate need S&R evacuation.

The function scheduleSR models how Search and Rescue (S&R) teams arrive and are allocated. When called, the function first performs a timeout between 15 and 20 minutes, representing the time it takes for firefighters to arrive at the scene and don their PPE. Depending on the S&R resource level—categorized as "Low," "Medium," or "High"—the number of available S&R teams is set to 2, 3, or 4, respectively. After this initial setup, the function waits for an additional period, which is a random time between 15 to 20 minutes. Then, it adds extra S&R teams: 1 extra team for "Low," 1 for "Medium," and 2 for "High," updating the total number of teams to 3, 4, or 6, respectively.

The Search and Rescue (S&R) activity is conceptualized as a procedure that employs S&R teams as a key resource for evacuating individuals who are unable to self-evacuate. This operation is represented by a time delay, which is a sum of the inter arrival rate and an added stochastic variation. The order in which victims are evacuated is randomized when pretriage is not carried out. When pretriage is performed, the evacuation happens in order of ascending triage category. There are no T3s in the pre-triage segment, as they have already left the station.

8. Triage algorithm

The triage category is a value of the patient object. It is set at patient initialization but is dynamic and can be changed at specific locations in the medical response chain. In this simulation, there are designated triage teams at the CCP, FMP and at the ED arrival. The triage category is reassessed before transport is started to ensure that the receiving hospital has adequate capacity to receive this victim. Triage at a decontamination location happens before decontamination at the CCP in strategy 1 and the ED in strategy 2 and 3.

When passing a triage point in the simulator the setTriage method is called to change the triage level of the patient object. The setTriage function combines the outcomes of both the setTraumaTriage and setChemicalTriage functions to determine a final triage level for the patient. It employs the min function of both categories in the same sense as described above.

The setTraumaTriage function aims to approximate the SALT (Sort, Assess, Lifesaving Interventions, Treatment and/or Transport) triage algorithm for trauma cases. First the current Simedis score is calculated based on the on the physical injuries only. A Simedis score of 17 or less is used as a surrogate for patients who are unable to follow commands, are in respiratory distress, have unpalpable peripheral pulsations, or have uncontrolled hemorrhage. Patients meeting these criteria are given a triage level of 1 (immediate intervention required). Conversely, patients with a Simedis score greater than 17 and who are mobile are triaged as level 3 (minor interventions required). Victims with a Simedis score greater than 17 but who are not mobile are triaged as level 2 (delayed intervention required). We considered using the AIS score to assess the “minor injuries only” criterium of the SALT method but failed to reach a consensus of the value to be used as a cutoff. We therefore used the assumption that minor injuries don’t impede mobility, while non-minor injuries do. Another limitation of the current implementation is that SALT adds a final category of expectant victims who are unlikely to survive given the current resources. This is not implemented in this version of the simulator, as this is more of an ethical question than a logistical one, as well as the limited number of victims as when compared to the rather large treatment capacity of the hospitals in the region.

The setChemicalTriage function assesses the severity of a patient's chemical exposure. The function determines the chemical Simedis score at the time of calculation based on the injury profile, and calculates the triage method accordingly. Patients with a score of 18 or above are classified as triage level 3, while those with a score between 13 and 18 are classified as level 2 (urgent but not immediate intervention required). Scores below 13 result in a triage level of 1.

9. Treatment functions

There are four different treatment types used in the simulation:

- Treatment type 1: This represents the FMP treatment. The c parameter gets increased by 0.02, increasing the survival time and decreasing speed of deterioration. This represents the MMT treatment and leads to a rise in SS and an increase in survival time of 25 to 30% depending on level of injury (with worse injuries resulting in a shorter stabilization period).
- Treatment type 2: This is the in-ambulance treatment. The c parameter gets increased by 0.03 in the case of MMT supervision and by 0.01 in the case of EMT supervision. This increase in survival time is only roughly 20%. It is important to note that the victim can still die from their injuries when receiving this treatment.
- Treatment type 3: This is the definitive (lifesaving) treatment and is only applied in-hospital. This is the only treatment that stops the victim from dying and changes the victim’s health state by changing the gamma parameter of the generalized Gompertz function to 0.2. This results in an evolution of the SS to plateau around 17 to 18.
- Treatment type 4: This is the AMS treatment, which reduces the IP level by one and does not change the traumatic health state evolution.

Treatment time of treatment type 1, 2 and 3 consider the patient's triage level, the presence of chemical exposure, and the severity of physical trauma (represented by the ISS). Treatment times for type 4 treatment are based on experimental data gathered during an exercise [4]. Treatment type 1 (EMT), 2 (MMT), and 4 (AMS) are exclusive to prehospital services, while Treatment type 3 is exclusive to the hospitals. The treatment time varies based on the patient's triage level and the severity of their condition. If strategy 2 or 3 are in place, the treatment time is increased by 50% to account for the additional complexity that PPE-gear adds to medical interventions.

The pseudocode of the treatment time calculation algorithm is described below.

*Treatment time starts at 0:*

*In the case of Treatment Type 1 (EMT) and Treatment Type 2 (MMT):*

- *Triage Level 3:*
  - *Chemical exposure adds 2 minutes.*
  - *Trauma adds 5 minutes.*
- *Triage Level 2:*
  - *Chemical exposure adds 5 minutes.*
  - *Trauma adds 10 minutes.*
- *Triage Level 1:*
  - *Chemical exposure adds 10 minutes.*
  - *Trauma adds 10 minutes.*

*In the case of Hospital Treatment:*

- *Triage Level 3:*
  - *Chemical exposure adds 10 minutes.*
  - *Trauma adds 10 minutes.*
- *Triage Levels 2 and 1:*
  - *Chemical exposure adds 15 minutes.*
  - *Trauma adds a base of 10 minutes plus an additional time calculated as the ceiling of the ISS divided by 3.*

Finally, stochastic variation is added to the calculated treatment time to add variability (and realism).

A more detailed explanation on the rationale and effects of this treatment model can also be found in Benhassine et al [3].

References

1. Faugier L, Marinus BG, Bosschaerts W, Laboureur D, Limam K (2021) CFD model to assess parameters influencing piston wind in a subway tunnel and station. J Phys: Conf Ser 2042:012076. https://doi.org/10.1088/1742-6596/2042/1/012076

2. Hosseini M, Madani H, Shahriar K (2022) CFD-based Modeling of Sarin Gas Dispersion in a Subway Station–A Hypothetical Scenario. Journal of Mining and Environment 13:. https://doi.org/10.22044/jme.2022.11604.2150

3. Benhassine M, De Rouck R, Debacker M, Hubloue I, Dhondt E, Van Utterbeeck F (2023) Simulating Victim Health State Evolution from Physical and Chemical Injuries in Mass Casualty Incidents. New Trends in Computer Sciences 1:113–25. https://doi.org/10.3846/ntcs.2023.19458

4. De Rouck R, Debacker M, Kneuts R, Van Hove SJL, Vaes J, Van Droogenbroeck P, Hubloue I (2019) Results of an in-hospital CBRN decontamination and stabilization exercise
