# Supplementary material for: Optimizing Medical Care during a Nerve Agent Mass Casualty Incident Using Computer Simulation
Source: J Med Syst. 2024 Sep 5;48(1):82. doi: 10.1007/s10916-024-02094-8 (PMC11377464; doi:10.1007/s10916-024-02094-8)
Supplement: Supplementary file 3 — Supplementary Material 3 [file 10916_2024_2094_MOESM3_ESM.docx]

Appendix 3: List of Abbreviations

Supplementary information to the article ‘Optimizing Medical Care during a Nerve Agent Mass Casualty Incident using Computer Simulation’ in Journal of Medical Systems

**Authors:**

De Rouck Ruben, MD (1)

Mehdi Benhassine, PhD (2)

Debacker Michel, MD (1)

Van Utterbeeck Filip, PhD (2)

Dhondt Erwin, MD (3)

Hubloue Ives, MD, PhD (1)

Corresponding Author: Ruben De Rouck – [ruben.de.rouck@vub.be](mailto:ruben.de.rouck@vub.be)

**Author Affiliations**

1. Research Group on Emergency and Disaster Medicine, Vrije Universiteit Brussel, Laarbeeklaan 103, 1090 Jette, Belgium
2. Department of Mathematics, Royal Military Academy, Renaissancelaan 30, 1000 Brussels, Belgium
3. DO Consultancy, Brussels, Belgium

Appendix 3: List of abbreviations used.

**AMS** - Advanced Medical Stabilization

**BITC** - Brussels Intercommunal Transport Company

**BLS** - Basic Life Support

**CBRNe** - Chemical, Biological, Radiological, Nuclear, and Explosive

**CCP** - Casualty Collection Point

**CWA** - Chemical Warfare Agent

**ED** - Emergency Department

**EMT** - Emergency Medical Technician

**EMS** - Emergency Medical Services

**FMP** - Forward Medical Post

**GB** - Sarin Gas (Chemical Formula)

**GCS** - Glasgow Coma Scale

**HAZMAT** - Hazardous Materials

**HC112** - National Emergency Dispatch Center (Specific to Belgium)

**IP** – Injury Profile

**ISS** - Injury Severity Score

**MCI** - Mass Casualty Incident

**MMT** - Mobile Medical Team

**NATO** - North Atlantic Treaty Organization

**PIT** - Paramedical Intervention Team

**PPE** - Personal Protective Equipment

**SALT** - Sort, Assess, Life saving interventions, Transport/treatment

**S&R** - Search and Rescue

**ScR** - Scoop and Run

**SS** – Simedis score

**StP** - Stay and Play

**T1, T2, T3** - Triage Categories (T1 being most severe, T3 least severe)
